# Deep learning-based selection of human sperm with high DNA integrity

**DOI:** 10.1038/s42003-019-0491-6

**Published:** 2019-07-03

**Authors:** Christopher McCallum, Jason Riordon, Yihe Wang, Tian Kong, Jae Bem You, Scott Sanner, Alexander Lagunov, Thomas G. Hannam, Keith Jarvi, David Sinton

**Affiliations:** 1 Department of Mechanical and Industrial Engineering, University of Toronto, 5 King’s College Road, Toronto, ON Canada M5S 3G8; 2 Hannam Fertility Centre, 160 Bloor St. East, Toronto, ON Canada M4W 3R2; 3 Department of Surgery, Division of Urology, Mount Sinai Hospital, University of Toronto, 60 Murray Street, 6th Floor, Toronto, ON Canada M5T 3L9

**Keywords:** Reproductive biology, Machine learning, Biotechnology, DNA damage and repair

## Abstract

Despite the importance of sperm DNA to human reproduction, currently no method exists to assess individual sperm DNA quality prior to clinical selection. Traditionally, skilled clinicians select sperm based on a variety of morphological and motility criteria, but without direct knowledge of their DNA cargo. Here, we show how a deep convolutional neural network can be trained on a collection of ~1000 sperm cells of known DNA quality, to predict DNA quality from brightfield images alone. Our results demonstrate moderate correlation (bivariate correlation ~0.43) between a sperm cell image and DNA quality and the ability to identify higher DNA integrity cells relative to the median. This deep learning selection process is directly compatible with current, manual microscopy-based sperm selection and could assist clinicians, by providing rapid DNA quality predictions (under 10 ms per cell) and sperm selection within the 86^th^ percentile from a given sample.

## Abstract (web summary)

Christopher McCallum et al. present a deep learning-based method for predicting DNA quality of individual human sperm from images. The method could be used for selecting sperm for assisted reproduction techniques, such as intracytoplasmic sperm injection.

## Introduction

Male infertility is a growing global health concern, with ~30% of infertility cases caused solely by male-factor infertility^1^. In certain cases of poor sperm health, assisted reproduction technologies (ARTs), such as intracytoplasmic sperm injection (ICSI), are employed, for which single sperm cells must be chosen from a population of ~10^8^ cells^2^. When selecting sperm cells for ICSI, clinicians rely on visual morphology criteria, such as sperm head size, and head, tail, and mid-piece shape according to the guidelines from the *World Health Organization* (WHO)^2^, after pre-screening for healthy cells (i.e., via density gradient and swim-up)^2,3^. While most clinicians view cells at moderate magnification (×40), high-magnification imaging (×63–100) of individual cells has proven useful^4^ to gain further insight into the morphological features mentioned above^5^. This method, intracytoplasmic morphologically selected sperm injection (IMSI), uses high-magnification microscopy and significantly improves blastocyst development, implantation, and pregnancy rates^4,6^. In addition, one group has developed automated IMSI for research purposes^7^. Notably, all single-cell selection methods to date depend solely on visual inspection using WHO morphology criteria as a guide to choose the best sperm cell^8–16^. Such an assessment relies heavily on the subjective choice of the clinician, and only accounts for externally observable features. In addition to human subjectivity, individual sperm inspection is ultimately low throughput, typically requiring inspection of tens of cells from a sample of tens of millions.

Deep learning is emerging as a preferred means of accomplishing visual inspection, classification, and selection tasks in a wide variety of applications in health and other sectors. The most common image analysis methods utilize deep convolutional neural networks (CNNs), with applications ranging from wild animal detection^17^ to cellular classification^18–20^ and tracking^21,22^, microscopy image enhancement^23^, biotechnology applications in microfluidics^24^, as well as for cancer and other disease diagnostics^25–31^. Deep learning has been employed to predict lineage choice in hematopoietic progenitors, solving the difficult problem of predicting objective, internal cell metrics from bright-field images^32^. In addition, deep learning was applied to label-free cell DNA analysis of human T cells via flow cytometry^33^, as well as to automated sorting of microalgal and human cells based on fluorescence and bright-field imaging^34^. In the fertility field, some groups have applied machine learning to classify sperm cells based on manually extracted features^10,13,16,35^ or via image-patch-based dictionary models^15^. While these approaches show promise, the algorithms were trained with a morphology metric determined by a human expert and lacked a quantitative objective sperm quality metric. With a human in the loop, these approaches fail to take advantage of a central advantage of deep learning, that is, the ability to learn from the sperm image data afresh, without the constraints of historical morphology evaluation practices.

DNA integrity is a quantitative, objective sperm quality metric that has been demonstrated to correlate with live birth outcomes^36^, making it well-suited for the training of deep-learning models. To objectively quantify sperm cell DNA integrity, clinicians employ various DNA integrity assays such as the sperm chromatin structure assay (SCSA), the acridine orange (AO) test, and single-cell gel electrophoresis (or Comet assay) which are easily quantified and provide standardized metrics for predicting male fertility^36,37^. Although useful as a diagnostic tool to assess whole-population male fertility potential, these DNA analyses cannot be employed in sperm selection because the fixing and staining procedures compromise cell viability, either by introducing dye into the cell nucleus or by fully lysing the cell. In a clinic, cell images are the only non-intrusive data-rich source of cellular information. Recently, we demonstrated a method to predict DNA quality based on morphological parameters extracted from bright-field images^38^, and we posit that a deep-learning model could instead assess images directly, without requiring pre-extraction of features. Thus, similar to current clinicians, the algorithm must take cell appearance as input, and make an objective sperm quality determination (i.e., based on DNA quality), in real time.

In this paper, we present a deep-learning-based method for ranking sperm according to sperm quality using DFI-labeled bright-field images, thus enabling selection of high-quality sperm for ICSI. Our method utilizes a deep CNN trained to predict sperm quality using the objective metric of individual cell DNA Fragmentation Index (DFI^37^, distinct from population-level %DFI) using only raw, label-free, sperm cell images. To train the neural network, we employed an in-house set of 1064 images of individual sperm cells of known DNA integrity. Our results demonstrate not only correlation between a cell image and the DNA integrity (with bivariate correlation ~0.43), but also the ability of our model to distinguish higher DNA integrity cells relative to the median with statistical significance. The trained model can assess an input sperm image and provide a DNA integrity prediction in under 10 ms, thus in principle enabling the rapid and consistent selection of high DNA integrity cells from a given sample.

## Results

### Predicting DNA integrity of unseen cells

**Fig. 1:**
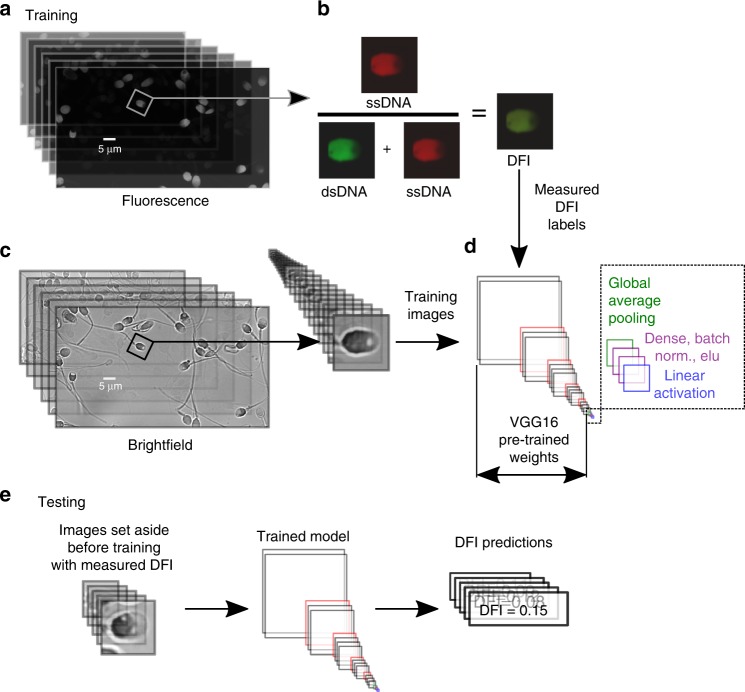
Experimental and modeling schematic. We illustrate the extraction of individual (**a**) fluorescence images to calculate the (**b**) DNA fragmentation index (DFI), as well as extraction of sperm head image from (**c**) bright-field microscopy images, which were used to train the (**d**) deep convolutional neural network. DFI was found using the acridine orange (AO) test^39,37^ (with brief details given in Methods and full details in Wang et al.^38^) and calculated as the ratio of red fluorescence (from presence of single-stranded DNA, ssDNA) to the sum of red and green fluorescence (from double-stranded DNA, dsDNA). The bright-field image was then labeled with the DFI value to train the model. The VGG16 network was modified by appending global average pooling and two dense layers with batch normalization and exponential linear unit (ELU) activation functions, after which linear activation was applied to condense the result to a single scalar value (DFI). **e** Once the model was trained, we fed images not used in training (but with measured DFI) and predicted the DFI, thereby yielding the generalizability of the model to unseen images

**Fig. 2:**
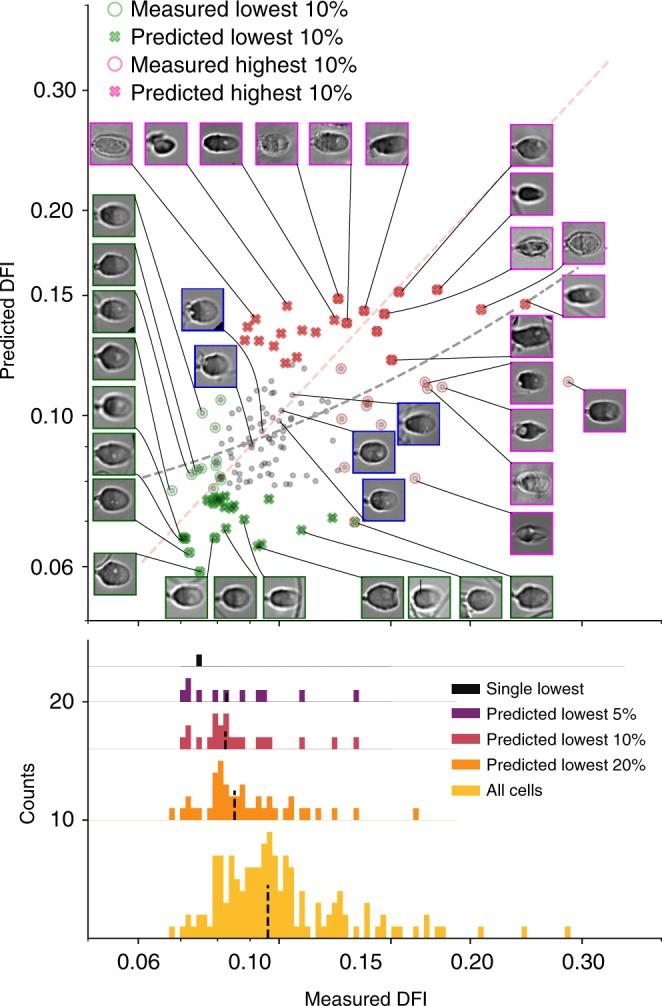
Results for the test set (20% of all cells, sampled evenly over all donors) show actual DFI versus predicted DFI (red dashed line shows actual = predicted for reference), as well as four highlighted groups: the actual-lowest 10% (green circles), predicted-lowest 10% (green ×’s), actual-highest 10% (magenta circles), and predicted-highest 10% (magenta ×’s). The blue-bordered images represent cells that the model predicted the most accurately. In general, the model performs better with fewer obstructions to the sperm head, fewer background features, and for more regularly shaped sperm heads

We trained a deep CNN to predict single-cell DFI as outlined in Fig. 1 using 1064 bright-field sperm cell images (with corresponding measured DFI) from six healthy donors (*N*_1_ = 150, *N*_2_ = 111, *N*_3_ = 89, *N*_4_ = 73, *N*_5_ = 134, *N*_6_ = 507) and found significant correlation (mean *R* ~0.43, *p* < 0.01) between actual and predicted DFI. First, considering all sperm images as a single dataset, we randomly segmented the labeled data into training (60%), validation (20%), and testing (20%) groups. After training and optimization (discussed in Methods section), the model evaluated the testing set, the results of which are shown in Fig. 2. We present the actual DFI versus the predicted DFI, highlighting example cell images from five groups of interest—the 10% predicted-lowest DFI and 10% actual-lowest DFI (green), the predicted-highest and actual-highest 10% (magenta), as well as example well-predicted median cells.

Comparing the median of both the predicted-lowest and predicted-highest groups with the population median indicates that the model quite capably distinguishes between the highest and lowest DNA integrity cells (DFI has an inverse correlation with DNA integrity, such that a high-quality sperm cell has low DFI and high DNA integrity). Given these predicted values in a clinical setting, one would select the cells with the predicted-lowest DFI. Here the single predicted-lowest DFI cell would be the 6th actual-lowest DFI cell out of this cohort of 213 never-before-seen images, representing selection of the 97th percentile. Also, between the predicted-lowest and actual-lowest DFI sets, we observe a significant overlap (with nine cells in common; *p* = 1.95e−5), which signifies that, clinically, when selecting the lowest 10% of cells, this set would contain nine of the actual-lowest DFI cells. In addition, the median of the lowest 10% predicted DFI sperm is at the 86th percentile, which, if selected by a clinician, would yield a sufficiently enriched sample to expect improvement in ICSI fertility outcomes^36^. This tool predicts an internal sperm DNA quality metric, otherwise unknown to a clinician, with reasonable accuracy and without damaging the cell.

### Testing model on sperm cells from individual donors

In the above model, the data for testing were isolated via random stratification over the six donors. In a clinical context, however, a model would be trained on some number of donors or patients, and then be applied to a fresh sample from a patient never previously studied. This clinical reality motivates an alternative training protocol, specifically, training with sperm from five of our six donors and reserving one of the donors entirely for test.

**Fig. 3:**
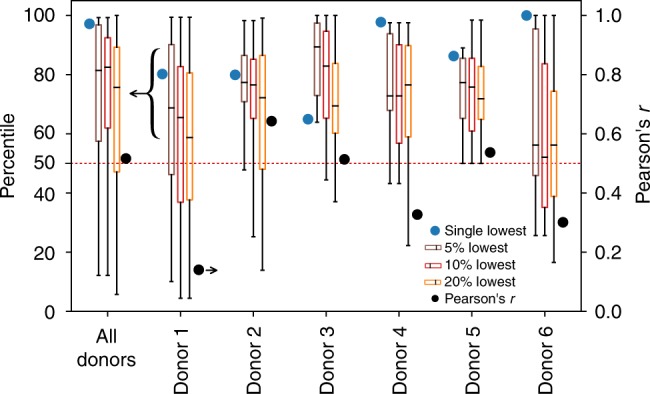
Percentile enrichment and Pearson’s *r*. Here, we highlight the percentile enrichment when sampling over all donors and for each individual donor, as well as overall Pearson’s *r* bivariate correlation (for which all *p*-values < 0.01) for each test set. The percentile enrichment shows the quartiles and extrema of the predicted-lowest DFI cells when selecting different proportions of the predicted-lowest cells. The average (mean) percentiles are 83, 74, 83, and 68, for the single, 5%, 10%, and 20%-lowest, respectively, highlighting the power of the model to enrich the sample. All values are given in Supplementary Table 1

We trained networks in this manner for each donor, isolating one of each of the donors in each case to be the test set. The resulting percentile enrichment based on predicted DFI and Pearson’s *r* results are shown in Fig. 3 (with all statistical values given in Supplementary Table 1). The available training set size was similar for Donors 1–5 (731–793), enabling direct comparison. Testing on Donor 6 is included, although due to the smaller training set available in that case (446 images), the model performed poorer (with bivariate correlation of 0.14 relative to 0.47 average across Donors 1–5). The percentile enrichment is calculated as the percent of cells with a higher DFI relative to a given cell, and directly translates to the level of enrichment in DNA integrity that a clinician would achieve if they chose the predicted-lowest DFI cell. For example, when a model trained on all donors except Donor 6, was applied to predict the DNA integrity of Donor 6, the selected best sperm (of 134) was the actual top-ranked sperm (100th percentile). Likewise, when applied to Donor 4, the top predicted sperm was the 98th percentile cell. The results of all donor-isolation combinations vary, as shown in Fig. 3, with the best predicted sperm being, on average, the 84th percentile sperm in terms of measured DNA integrity. In addition, the Pearson’s *r* values (with a mean of 0.43) indicate a high degree of linear correlation (*p* < 0.01 for all cases) between the model-predicted and measured DFI values.

### Enabling subpopulation DNA integrity enrichment

**Fig. 4:**
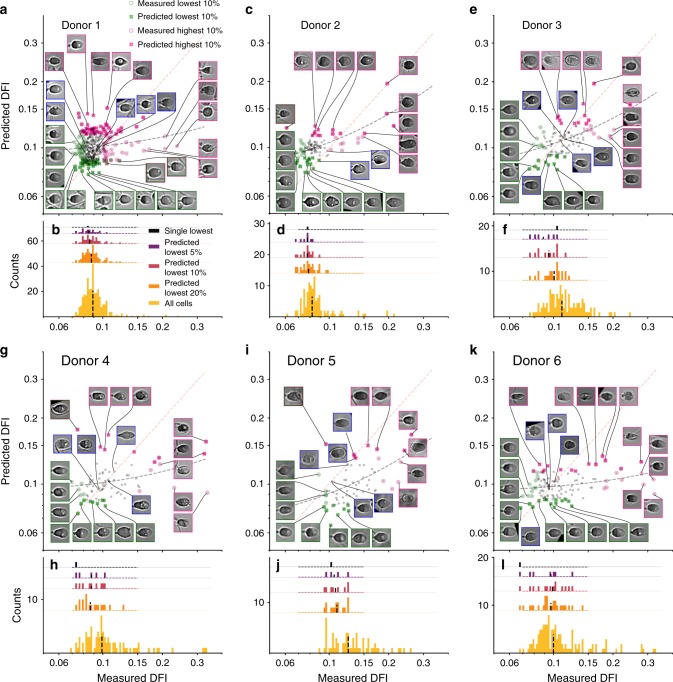
Predicted versus measured DFI when testing model on individual donors. As in Fig. 2, the (**a**, **c**, **e**, **g**, **i**, **k**) predicted versus measured DFI for Donors 1–6, respectively, as well as the (**b**, **d**, **f**, **h**, **j**, **l**) enrichment when selecting a certain percentage of the best (lowest-predicted DFI) cells. Overall, the model-predicted-lowest cells agree with the actual-lowest DFI cells, especially as the size of the lowest-predicted group is decreased

A potential use for our approach would be to use the model to screen a sample for a subpopulation of very good ICSI candidates. In such cases, a model could be used to select a group of top sperm from which human clinicians would then select individual sperm for ICSI. We tested the model at selecting the top 5, 10, and 20% of cells with the metric of achieved percentile enrichment, as shown in Fig. 3, for which we achieve median percentiles of 74, 73, and 68, respectively. To further visualize the range of cells present in the different percentage groups, Fig. 4 shows the predicted versus measured DFI when the final test set is composed of Donors 1–6, as well as the entire measured DFI range for each donor with an overlay of the measured DFI of the single predicted-lowest cell and predicted-lowest 5, 10, and 20% cells. Most of the predicted-lowest DFI cells agree well with the actual-lowest DFI cells since the median is always greater than the 50th percentile. Therefore, according to these conditions, a clinician could select from a pool of model-predicted top 5% DNA integrity cells with the expectation that the median in this pool is the 74th percentile (±12%, s.d.). The clinician could then apply their current norms of sperm evaluation (such as motility and morphology) for clinical ICSI. In that final selection process, the clinician could also have the ranking of individual cells within the top 5% predicted pool, if desired.

### Model limitations

Poorly predicted cells are principally a result of debris present in the image near the sperm head or poorer-quality contrast images. Probing individual cell images, Figs. 2 and 4 highlight the successes and failures of the DFI predictions. The bottom-left group of images represent the greatest successes of the model, the overlap in the predicted-lowest and actual-lowest sets. Ideally, the model would rank all cells in order in terms of DFI, but predicting the lowest DFI cells is of much greater clinical utility, meaning accurate predictions in this region are paramount to model success. More importantly, the greatest error is found for higher DFI (magenta-outlined) cells, which are largely under-predicted. Underpredicting these moderate-DFI, normal-morphology cells (i.e., overpredicting quality) could pose a problem for clinicians, though only a few such cases are present here (lowest insets in Figs. 2 and 4). In addition, certain cases show considerable background debris and sperm tails in the field of view that are likely to have biased the prediction. Omitting poor image-quality cells improves overall DFI prediction as mean percentile enrichment rank across 5, 10, and 20% groups increases by 5.3%, and bivariate correlation increases by 6.9% (as given in Supplementary Table 2). In this subtest, the poor-quality images were removed manually, but in practice a screening algorithm could be trained to remove images including, for instance, extraneous tail components. Last, testing on a dataset imaged four months after the original set (Supplementary Figure 2) showed limited correlation, highlighting the importance of data imaged under varying conditions.

### Highlighting features important for predicting DFI

**Fig. 5:**
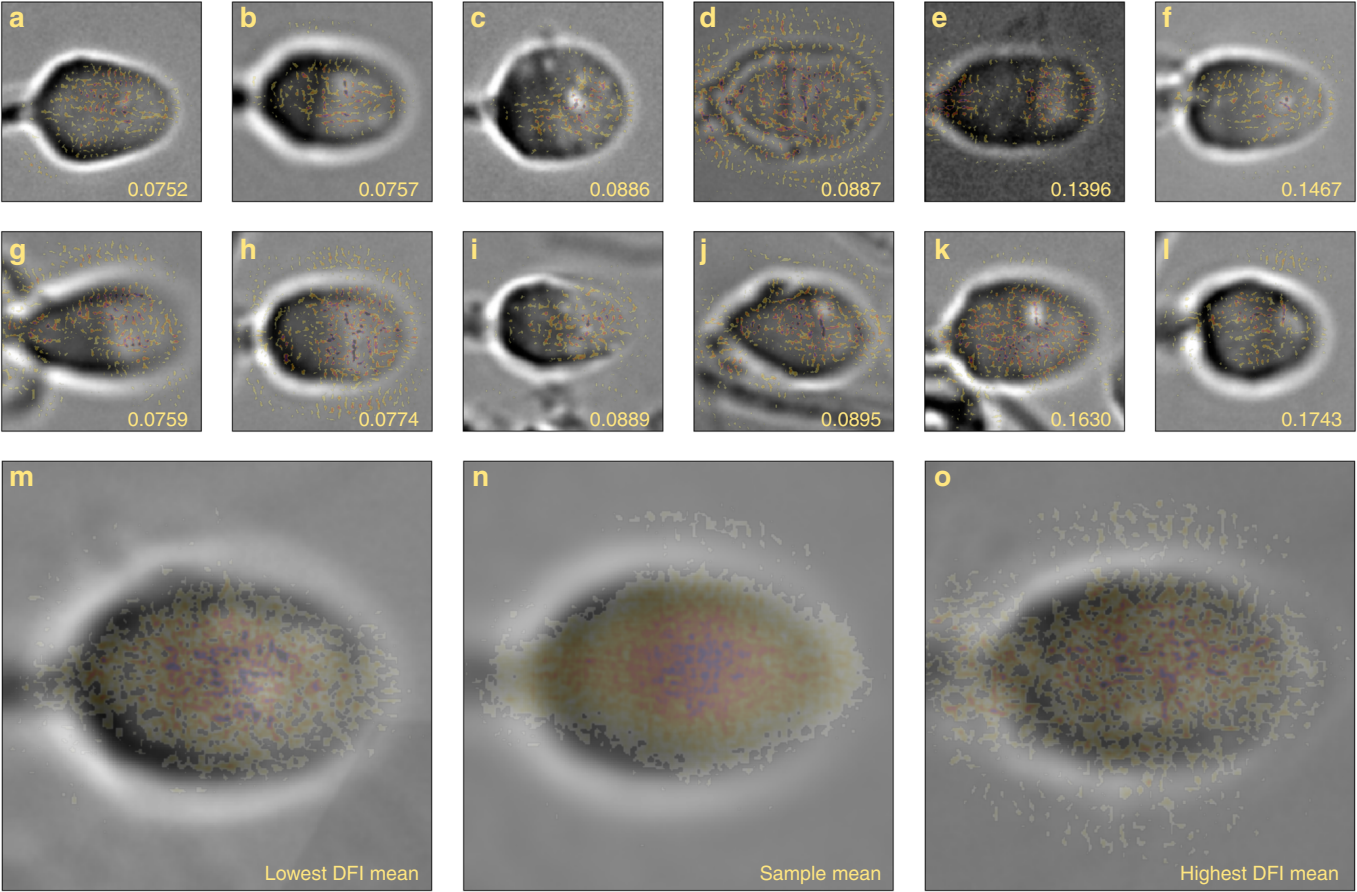
Bright-field cells images with saliency map overlay. The saliency map (color overlay) computes the gradient of the output category (DFI, given in bottom right corner) with respect to the input image (gray-scale background images), which highlights important features determined by the model. Specifically, we show examples of the (**a**, **b**, **g**, **h**) lowest 10% DFI cells with (**m**) the mean bright-field image of these cells and mean of the saliency maps overlaid, the (**c**, **d**, **i**, **j**) median 10% DFI cells with (**n**) mean bright field and saliency, and the (**e**, **f**, **k**, **l**) highest 10% DFI cells with (**o**) mean bright field and saliency for highest DFI cells. The dark intensity regions of the heat map indicate greater pixel importance. The model shows some background noise but primarily identifies internal features and places low value on undesirable features such as tails in the field of view that may not be associated with the sperm head of interest

Saliency maps are commonly employed to weigh the influence of pixels used by the model to make predictions based on individual image inputs (i.e., pixels that most strongly contribute to the class score)^30,40^. These saliency maps, shown in Fig. 5, illustrate that the model generally focuses on the internal features of the cell and largely disregards the background in determining the DFI. To some degree, though, the model does give weight to artifacts such as sperm tails, debris in the field of view, or background noise. Moreover, for low DFI cells, the heatmap is localized in the cell center at the intersection between the nucleus (left head region) and acrosome (right head region), while for high DFI cells, the nucleus and mid-piece pixels are more influential. When taking the average of all saliency maps (Fig. 5o), it is apparent that high importance is given to the intersection between the nucleus and the acrosome. The influence of the model in this region reflects the biological importance of the nucleus, which contains the DNA cargo, and the acrosome, which can contain abnormalities such as vacuoles. Furthermore, when analyzing specific cells with vacuoles (Fig. 5d, g, k, l), it is apparent that substantial emphasis was given to these regions, meaning the presence of vacuoles played a role in DFI prediction.

### Differentiating between cells of similar morphology

**Fig. 6:**
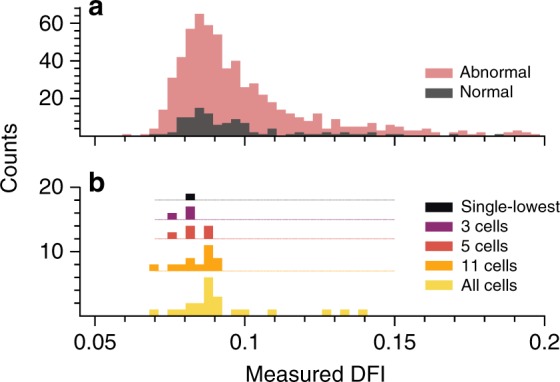
Normal versus abnormal DFI distribution and sub-group measured DFI. The histograms display DFI variation (**a**) over the entire dataset showing clinician classification of cells into abnormal and normal groups and (**b**) for one representative test set after training a model on only normal cells. The DFI values were truncated at 0.2 for ease of viewing due to the sparsity at higher DFI. **a** Performing the *t*-test for the medians of the normal and abnormal populations yields a *p*-value of 0.41, signifying that the medians are statistically the same. **b** The different cell quantities (i.e., 3 cells, 5 cells, etc.) illustrate the actual DFI values for the specific group of the predicted-lowest DFI cells

A skilled clinician analyzed our cell images directly, with no knowledge of the individual cell DFIs, and classified each sperm as normal or abnormal. The clinician-selected normal group reflected the overall sperm quality distribution, as shown in Fig. 6. No difference in median DFI was found between the clinician-selected normal group and the population (*p* = 0.41). While not a comprehensive assessment of clinical ability, this result implies that the ability of the model to sort sperm images with respect to DFI is not replicated in current clinical selection.

To test the model viability in differentiating between only normal cells, we trained a model using only the normal cell subset with a training size of 84 and validation size of 22 images. After fivefold cross validation, we determined the model has lower success (71st percentile enrichment and Pearson’s *r* of 0.48) relative to choosing the best cells from the entire population of over 1000 cells, as shown in Table 1. Nevertheless, the model successfully distinguished between cells of similar morphology and chose the best, high DNA integrity cells. Given the large number of cells available for selection, the model and clinician are thus complementary. The model can assess a large number of cells and select a subset of sperm with high DNA integrity, from which the expert can choose a single sperm based on the current variety of metrics, clinical norms, and individual skill. Alternatively, given the complementary nature of our prediction, our method is also immediately useful in informing last-stage selection, where a clinician is tasked with choosing among identical-looking normal sperm candidates, and would benefit from deep-learning-based insight.

**Table 1:** Percentile enrichment, Pearson’s *r* (with corresponding *p*-values), and MAE for a model that only includes normal cells as determined by clinician assessment

k-Fold number	20% Predicted lowest					10% Predicted lowest					Lowest	Pearson’s *r*		MAE
	%	*p*	*n*	dof	*t*	%	*p*	*n*	dof	*t*	%	*r*	*p*	
1	81.8	2.3E−02	5	25	2.43	86.4	2.1E–03	3	23	3.48	81.8	0.456	3.3E−02	0.016
2	57.1	6.5E−01	5	24	0.47	76.2	3.6E−02	3	22	2.41	57.1	0.171	4.6E−01	0.015
3	85.7	1.4E−03	5	24	3.65	85.7	1.7E−03	3	22	3.61	81.0	0.615	3.0E−03	0.020
4	85.7	6.6E−03	5	24	2.97	81.0	2.2E−02	3	22	2.49	81.0	0.456	3.8E−02	0.012
5	71.4	4.2E−01	5	24	0.85	52.4	9.1E−01	3	22	0.13	52.4	0.700	4.1E−04	0.011
Mean	76.4					76.3					70.6	0.480		
s.d.	12.2					14.0					14.6	0.202		

## Discussion

Overall, this work indicates that sperm DNA integrity can be predicted from a sperm image alone through supervised training of a deep convolutional neural network. The successes of our proposed model on only six donors notwithstanding, building a clinical technology would require labeled sperm from 1000 s of patients and donors. Also, our model could be improved further by considering alternate scoring methods such as aneuploidy, motility, as well as other DNA quality metrics (e.g., COMET and TUNEL (terminal deoxynucleotidyl transferase dUTP nick end labeling)).

Furthermore, this deep-learning selection process is directly compatible with current, manual microscopy-based sperm selection and complementary to current clinical selection that does not select single sperm with high DNA integrity. This method would initially serve to complement existing analysis methods used by fertility clinicians, by allowing for real-time (10 ms per cell) differentiation between cells of varying DFI—and thus sample enrichment based on DFI—as cells are viewed by the clinician. The final selection decision would ultimately still fall to clinicians, but the additive power of deep learning would enable a more informed decision.

Certain challenges must be overcome to realize total clinical applicability, both regarding the model discussed and the technology required to implement the model. While IMSI—and thus high-magnification sperm cell imaging—increase overall pregnancy rates^4,6^, this approach requires ×100 magnification, which may not be compatible with clinical workflow. Nevertheless, new developments would allow for automated sperm imaging and tracking^41^, which would relieve much of the burden of clinicians and enable direct compatibility with our proposed model. Therefore, we believe that clinics will adapt to new protocols and technology once proven effective.

Moreover, the complementary role of deep learning and AI will no doubt transform the current health care system as health and data sciences converge^42–44^. Although initial applications in retinal imaging and bone-fracture detection have been FDA-approved^45^, broader implementation challenges currently exist, such as gaining patient trust, integrating AI systems into current workflow, and validating models across wide, heterogeneous populations^46^. Therefore, in the near future, deep-learning output will serve simply as statistical predictions to assist clinicians in interpreting medical data. Results here indicate that models have potential to excel at the fundamental task of single human sperm selection for artificial reproduction.

## Methods

### Sperm cell imaging protocol and dataset

We employed an in-house dataset of bright-field and fluorescence images—from acridine orange (AO) staining—obtained via ×100 (objective magnification) confocal microscopy, with full details reported elsewhere^38^. Briefly, a glass cover slide was treated with piranha solution (3:1 sulfuric acid to H_2_O_2_) for 30 min followed by immersion in 10% v/v APTES in acetone, rinsed with acetone, and then air dried. After heating the slide to 110 ℃ for 60 min and cooling it down to room temperature, the slide was treated with a solution of hyaluronic acid (HA), EDC-HCl, and NHS dissolved in MES buffer (stirred for 1 h) for 30 min to functionalize the surface and allow for sperm binding. The donor semen samples (frozen, purchased from ReproMed Ltd; all donors provided consent for research participation in accordance with regulations of the Assisted Human Reproduction Act) were thawed at 37 ℃, washed with pure sperm wash, centrifuged at 300 × *g* for 5 min with an additional wash, and then loaded into a custom PDMS reservoir on the HA-functionalized cover slip. The solution was then evaporated, after which the sperm cells were treated with TNE buffer and acid-detergent solution before AO was added to stain the cells (to detect single-stranded fragmented DNA and double-stranded DNA). Sperm were imaged immediately after staining under a spinning disk confocal microscope under a total magnification of ×100 with excitation wavelength of 488 nm and emission filters of 500–550 nm for green and 598–660 nm for red. Fluorescence images were captured first, after which bright-field images were obtained.

**Fig. 7:**
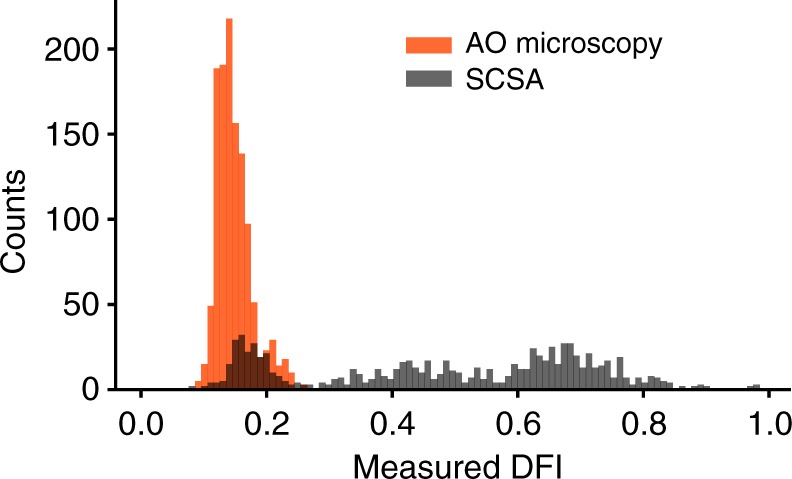
DFI histogram comparing recent single-cell DFI based on AO microscopy^38^ and traditional SCSA. A sample was split into two, and each half was analyzed independently via either method. Both methods yield DFI values based on AO intercalation, although AO microscopy does not capture higher DFI cells, due to differences in imaging, as well as the exclusion of debris, cell aggregates, and non-sperm species, none of which is excluded in traditional SCSA^5^

This staining protocol was consistent with the sperm chromatin structure assay (SCSA)^37^, considered the gold standard in DNA fragmentation measures^5^, although our specific imaging method varied slightly (the green emission bandwidth of the confocal microscope was 500–550 nm relative to 515–530 nm of SCSA), ultimately yielding DFI values shown in Fig. 7. Furthermore, we report individual cell DFI rather than the commonly specified %DFI, or proportion of damaged cells. Also, the proprietary flow cytometry normalization method does not allow for simple comparison with the arbitrary intensity from the microscopy technique. Overall, AO has proven to effectively highlight DNA fragmentation (a measure of DNA integrity) in sperm cells^36,39^. In this work we highlight our efforts to measure single-cell DFI and correlate this to the bright-field image, rather than measure population-level DNA fragmentation.

Each bright-field image and corresponding fluorescence images contained ~5 sperm cells per image which were manually cropped and rotated (via opencv-python v3.4.4) to select the individual sperm cell heads, yielding a final dataset of 1064 images across six healthy donors (*N*_1_ = 150, *N*_2_ = 111, *N*_3_ = 89, *N*_4_ = 73, *N*_5_ = 134, *N*_6_ = 507). The individual DFI values were calculated as the ratio of total area intensity of the single-stranded DNA fluorescence over the sum of the single-stranded and double-stranded total area fluorescence intensity, after background correcting the two fluorescence images. We also analyzed the bright-field intensity of each image and found very low correlation with sperm head intensity or background intensity with DFI, thus ensuring that the model cannot derive a false relationship based on fluorescence stain-based brightness of images themselves and DFI (as shown in Supplementary Fig. 1). Last, we trained a new model that allowed for free rotation of the input image and found similar correlation between measured and predicted DFI (shown in Supplementary Table 3) relative to the primary model that limits rotation to 10°, meaning that the rotation operation does not result in artificial correlation between the DFI and bright-field image.

### Deep-learning model architecture

We implemented a deep-learning model (with full architecture given in Supplementary Table 4) that employs the VGG16^47^ convolutional neural network (CNN) architecture pre-trained on the ImageNet^48^ database written in Python (v3.6) using Keras (v2.1.5)^49^ on top of TensorFlow (v1.8.0)^50^. After the last convolutional layer, we appended a global average pooling layer followed by two fully connected layers (of widths 502 and 667) with batch normalization and an exponential linear unit^51^ activation function. Last, to output a DFI value, we add a fully connected layer with linear activation with one output. This network, therefore, differs from most CNNs since it yields an unbounded real scalar instead of typical classification scores from a softmax layer. We train only our last appended layers, keeping the original VGG16 weights, until the validation mean-squared error ceases to decrease and also tested using mean-absolute error and 90% quantile regression^52^ loss functions. This method remains consistent with well-established transfer learning procedures^53,54^ that allow for rapid model training on new image sets (including medical images^30^ and small datasets^53,55^) built on the framework of powerful networks trained on generic images.

### Model training

The cropped cell images were originally 150 × 150 pixels, which were scaled up to 224 × 224 (according to VGG16 requirements) via bilinear interpolation. During training, 32 images were mini-batch processed with minor image augmentation allowing randomized rotation up to 10°, vertical and horizontal flipping, as well as vertical and horizontal shifting up to 5% to reduce overfitting and to artificially inflate the total number of training images. We trained the model using RMSprop optimization—finding similar performance with Adam optimization^56^—with a learning rate = 2.9 × 10^−5^ using a GeForce GTX1060 by NVIDIA.

### Bayesian optimization

**Fig. 8:**
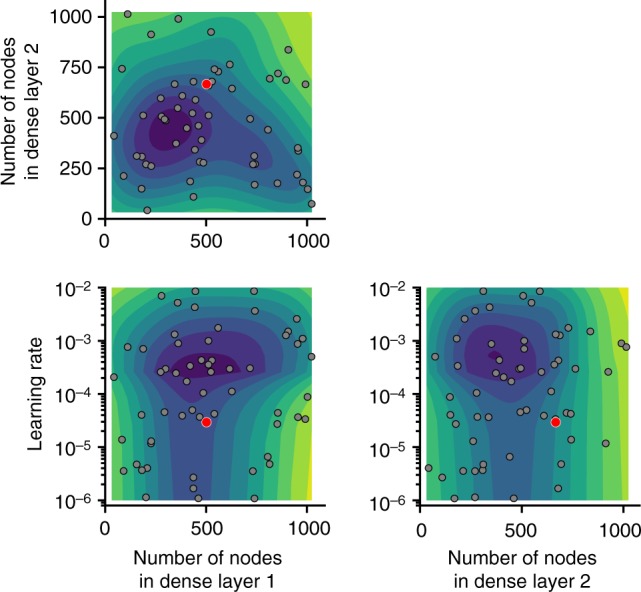
Results of Bayesian optimization using Gaussian processes, highlighting the influence of the number of nodes in final two fully connected layers and learning rate when optimizing for validation mean-squared error. The model was also optimized for activation function in the fully connected layers (ReLU, ELU, tanh), loss function (mean-squared error, mean-absolute error, and 90% quantile regression), as well as for batch normalization layers versus dropout layers (with different dropout rates), and, last, for Adam versus RMSprop optimizers. The optimized model (the model with the lowest error, as denoted by the red points) contained 502 nodes in the penultimate dense layer and 667 nodes in the final dense layer, used the ELU activation function and batch normalization between each dense layer, and was optimized via RMSprop for a learning rate of 2.9 × 10^−5^

Much of the success of our model was due to Bayesian optimization using Gaussian processes (gp_minimize function of scikit-optimize v0.4) to fine-tune model hyper-parameters (i.e., learning rate, number of dense nodes, activation function, loss function, and model optimizer). Figure 8 shows representative partial dependence when optimizing the number of nodes in the final two fully connected layers and the learning rate.

### Learning curve analysis

**Fig. 9:**
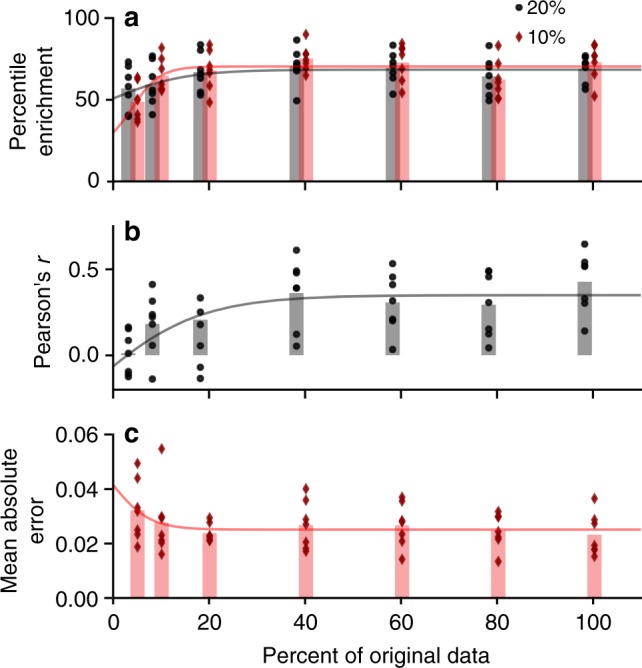
Learning curve analysis—model performance when including fewer training images—as given by **a** percentile enrichment, **b** Pearson’s *r*, and **c** mean-absolute error. Data points given performance of seven different test sets (for six donors and randomized), bars show the mean over the seven runs, and curve shows fitting of saturation curve. Given the trends and saturation at 100%, model performance is unlikely to increase simply with more training examples (more sperm cell images)

Given more data, would model performance increase? One would expect model performance to converge toward one value given infinite data, and as the amount of data is increased, performance saturates. This plateau was observed in our case, as shown in Fig. 9, when fitting a sigmoid function of the form $f\left( x \right) = \frac{a}{{1 + \exp \left( { - b\left( {x - x_0} \right)} \right)}} + c$. Therefore, given a greater number of sperm cell images, model performance would not be expected to improve substantially.

### Statistics and reproducibility

We analyzed 1064 bright-field sperm cell images (with corresponding measured DFI) from six healthy donors (*N*_1_ = 150, *N*_2_ = 111, *N*_3_ = 89, *N*_4_ = 73, *N*_5_ = 134, *N*_6_ = 507) for model training. The *t*-tests performed to analyze the difference in median DFI values utilized the independent two-sided *t*-test (from the stats package of SciPy v1.1.0) with unequal variances. We chose to analyze the median because of the log-normal distribution of the data. The *p*-values associated with each percentile indicate the significance in the difference between the subpopulation (5, 10, 20%) median and the total population median. The Pearson’s *r* analysis relied on SciPy as well to calculate the coefficient and *p*-value. The measurements were taken from distinct samples, not measured repeatedly.

The model performance remained consistent and reproducible when re-trained from scratch, with similar correlation and percentiles obtained for individual donors, as indirectly observed in the learning curve analysis. Such consistency is expected when training on the same images with the same model architecture. Given new image training data and different model architecture, the model weights and predictions may vary, but the performance outlined in this manuscript is indicative of general performance.

### Reporting summary

Further information on research design is available in the Nature Research Reporting Summary linked to this article.

## Supplementary information

Supplementary Information

Reporting Summary

## Data Availability

The datasets generated during and/or analyzed during the current study are available in the figshare repository^57^, https://figshare.com/articles/Deep_learning-based_selection_of_human_sperm_with_high_DNA_integrity/8124932.

## Code availability

All custom code was developed in Python (as described in the deep-learning model section) and is available via GitHub https://github.com/cmccallum08/Deep-learning-based-selection-of-human-sperm-with-high-DNA-integrity/tree/v1.1 and Zenodo^58^ https://zenodo.org/record/3238696. The repository includes cell cropping and DNA integrity calculations, the convolutional neural network model, and post-processing of model performance data.
