# Supplementary material for: Deep learning-based selection of human sperm with high DNA integrity
Source: Commun Biol. 2019 Jul 3;2:250. doi: 10.1038/s42003-019-0491-6 (PMC6610103; doi:10.1038/s42003-019-0491-6)
Supplement: Supplementary file 1 — Supplementary Information [file 42003_2019_491_MOESM1_ESM.pdf]

Supplementary Figures

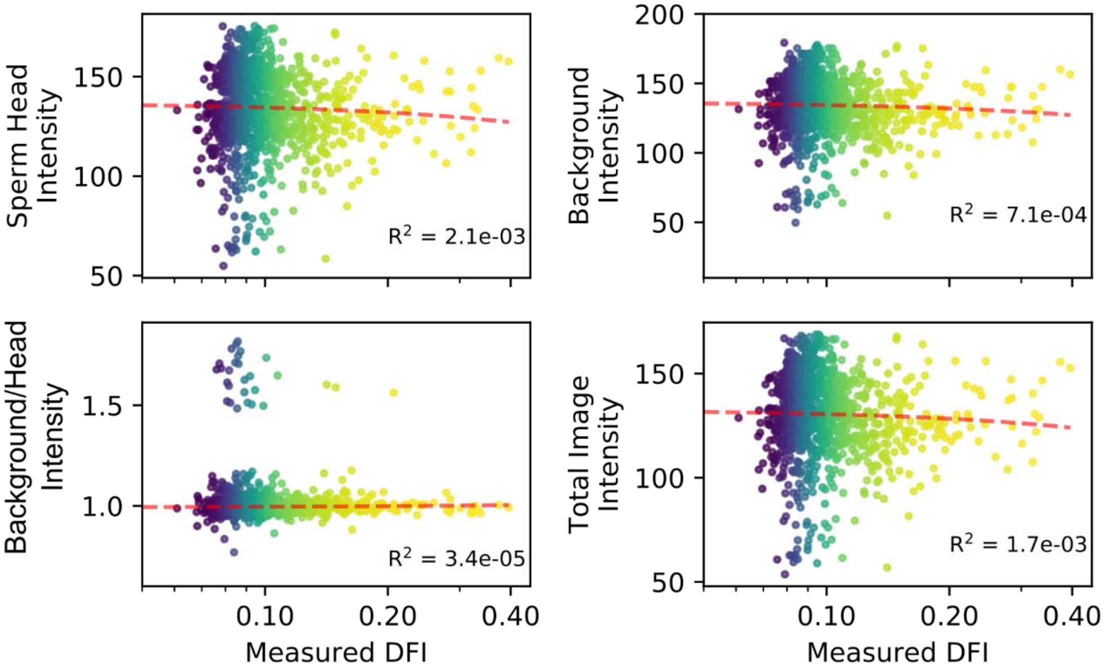

Supplementary Figure 1: Least-squared fitting to determine correlation and influence between brightfield image intensity and DFI. The cells were stained to determine DNA integrity, which could have affected the brightfield imaging (and thus the inputs to the CNN). Therefore, we performed a test to determine if correlation exists between the brightfield images and DFI, finding very low correlation between sperm head area-averaged intensity, brightfield image background intensity, ratio of head to background, and area-averaged total image intensity to show that the images themselves will not influence model results.

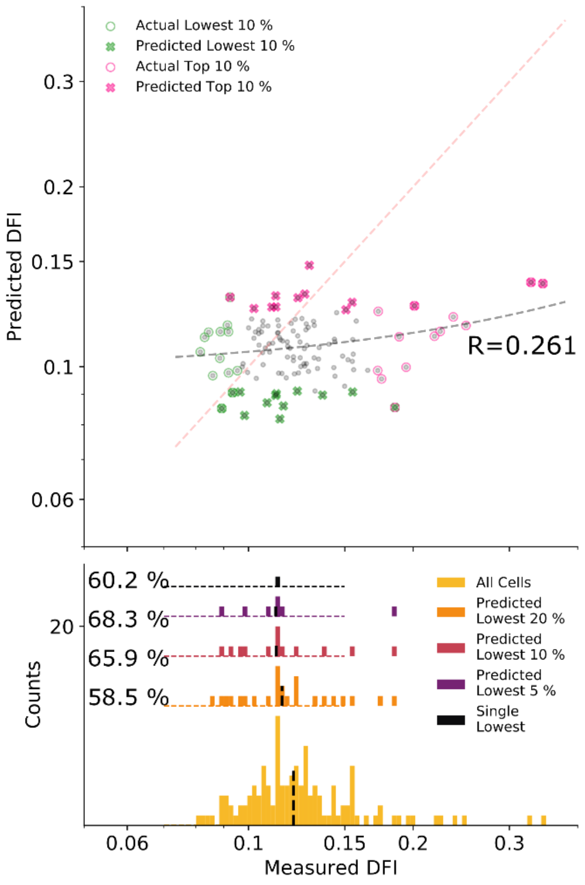

Supplementary Figure 2: Predicted versus measured DFI when testing model on a 7th donor that was trained on Donors 1-6. As in Figure 2, the predicted DFI is shown as well as the enrichment when selecting a certain percentage of the best (lowest predicted DFI) cells.

Supplementary Tables

Supplementary Table 1: Percentile enrichment, Pearson's *r*, and mean absolute error (MAE) for different test sets

Donor	20% predicted lowest					10% predicted lowest				
	%	p	n	dof	t	%	p	n	dof	t
**1**	58.7	1.2E-03	102	605	3.26	65.5	4.5E-03	51	554	2.90
**2**	72.2	7.9E-04	30	178	3.43	76.5	5.1E-05	15	163	4.24
**3**	69.4	7.5E-06	23	132	4.78	83.3	1.6E-05	12	121	5.21
**4**	76.5	2.6E-04	18	105	3.80	72.8	1.9E-04	9	96	3.97
**5**	71.9	1.2E-05	15	86	4.69	76.6	3.4E-04	8	79	4.06
**6**	56.2	7.8E-04	27	158	3.45	52.1	4.0E-03	14	145	3.03
**all**	75.7	7.2E-04	43	254	3.47	82.9	2.5E-04	22	233	3.93
**mean**	68.7					72.8				
**s.d.**	8.1					11.0				

Donor	5% predicted lowest					Single lowest	Pearson's r		MAE
	%	p	n	dof	t	%	r	p	
**1**	69.0	2.7E-04	26	529	3.92	80.2	0.140	9.6E-03	0.015
**2**	78.3	7.9E-05	8	156	4.35	79.9	0.643	9.6E-15	0.019

**3**	90.7	9.9E-04	6	115	4.91	64.9	0.514	1.3E-08	0.018
**4**	72.8	1.6E-03	5	92	3.69	97.8	0.327	2.9E-03	0.027
**5**	78.1	1.1E-02	4	75	3.43	86.3	0.537	4.8E-06	0.036
**6**	56.2	1.7E-02	7	138	2.81	100.0	0.301	8.0E-04	0.029
**all**	81.4	2.2E-02	11	222	2.55	97.2	0.517	6.5E-11	0.018
**mean**	75.2					86.6	0.425		0.023
**s.d.**	10.8					12.7	0.174		0.008

We show the percentile increase when comparing the total test set median with the median of a given percentage of the predicted-lowest DFI cells (and associated p-value based on the student t-test of the DFI rankings to compare the difference in median values) to determine how accurately the model predicts the lowest DFI cells (and thus the clinical application viability of our model), as well as overall Pearson's *r* bivariate correlation (and associated significance values), mean absolute error (MAE).

Supplementary Table 2: Percentile enrichment, Pearson's *r*, and mean absolute error (MAE) for different test sets after removing poor-quality images

Donor	20% predicted lowest					10% predicted lowest				
	%	p	n	dof	t	%	p	n	dof	t
1	54.2	5.9E-02	46	269	1.91	63.1	1.9E-03	23	246	3.29
2	72.2	1.5E-03	24	137	3.26	75.7	2.4E-03	12	125	3.27
3	72.3	8.1E-05	19	111	4.23	72.3	1.9E-03	10	102	3.46
4	72.8	1.9E-04	17	96	3.88	80.2	9.7E-05	9	88	4.18
5	81.3	3.4E-06	13	75	5.07	84.4	3.0E-04	7	69	4.33
6	66.1	2.7E-04	25	144	3.77	71.1	2.7E-03	13	132	3.29
all	85.0	7.8e-06	29	167	4.74	87.9	6.7e-06	15	153	5.19
mean	72.0					76.4				
s.d.	10.1					8.5				

Donor	5% predicted lowest					Single lowest	Pearson's r		MAE
	%	p	n	dof	t	%	r	p	
1	61.3	9.2E-02	12	235	1.80	56.9	0.284	1.5E-05	0.016
2	80.0	2.6E-04	6	119	4.37	83.5	0.558	9.6E-11	0.024
3	84.0	2.5E-04	5	97	5.15	66.0	0.521	7.3E-08	0.020
4	93.8	3.3E-03	5	84	3.63	97.5	0.383	4.1E-04	0.027
5	78.1	1.6E-03	4	66	4.21	84.4	0.554	2.1E-06	0.031
6	67.8	1.1E-03	7	126	3.97	100.0	0.383	1.5E-05	0.028
all	93.6	5.7e-09	8	146	7.63	97.9	0.502	2.7e-10	0.017
mean	79.8					83.7	0.455		0.023
s.d.	12.2					16.8	0.105		0.006

Supplementary Table 3: Percentile enrichment, Pearson's *r*, and mean absolute error (MAE) for different test sets when allowing for free rotation of input images.

Donor	20% predicted lowest					10% predicted lowest				
	%	p	n	dof	t	%	p	n	dof	t
1	55.5	1.5E-02	102	605	2.45	69.0	1.3E-02	51	554	2.55
2	70.4	5.2E-04	30	178	3.55	68.7	3.3E-03	15	163	3.06
3	69.4	1.1E-02	23	132	2.64	67.6	4.0E-02	12	121	2.22
4	61.7	8.9E-03	18	105	2.68	72.8	3.2E-03	9	96	3.16
5	70.3	1.6E-01	15	86	1.47	82.8	1.6E-03	8	79	3.68
6	56.2	1.4E-03	27	158	3.27	57.9	9.6E-03	14	145	2.73
all	75.7	9.1E-06	43	254	4.58	78.6	3.8E-06	22	233	4.93
mean	65.6					71.1				
s.d.	7.8					8.1				

Donor	5% predicted lowest					Single lowest	Pearson's r		MAE
	%	p	n	dof	t	%	r	p	
1	73.2	2.6E-02	26	529	2.33	96.2	0.293	3.9E-08	0.017
2	64.3	6.7E-02	8	156	1.95	87.3	0.559	8.6E-11	0.025
3	86.1	2.2E-02	6	115	2.96	34.4	0.464	4.2E-07	0.019
4	93.8	1.5E-02	5	92	2.94	97.8	0.346	1.6E-03	0.029
5	78.1	1.4E-02	4	75	3.32	90.4	0.430	3.9E-04	0.036
6	62.0	6.7E-02	7	138	2.08	60.2	0.180	4.8E-02	0.030
all	86.4	1.3E-09	11	222	7.07	98.6	0.535	1.0E-11	0.019
mean	77.7					80.7	0.401		0.025
s.d.	11.9					24.3	0.136		0.007

Supplementary Table 4: Complete deep learning model architecture

Layer	(type)	Output Shape	Param #
**input_1**	(InputLayer)	(None, 224, 224, 3)	0
**block1_conv1**	(Conv2D)	(None, 224, 224, 64)	1792
**block1_conv2**	(Conv2D)	(None, 224, 224, 64)	36928
**block1_pool**	(MaxPooling2D)	(None, 112, 112, 64)	0
**block2_conv1**	(Conv2D)	(None, 112, 112, 128)	73856
**block2_conv2**	(Conv2D)	(None, 112, 112, 128)	147584
**block2_pool**	(MaxPooling2D)	(None, 56, 56, 128)	0
**block3_conv1**	(Conv2D)	(None, 56, 56, 256)	295168
**block3_conv2**	(Conv2D)	(None, 56, 56, 256)	590080
**block3_conv3**	(Conv2D)	(None, 56, 56, 256)	590080
**block3_pool**	(MaxPooling2D)	(None, 28, 28, 256)	0
**block4_conv1**	(Conv2D)	(None, 28, 28, 512)	1180160
**block4_conv2**	(Conv2D)	(None, 28, 28, 512)	2359808
**block4_conv3**	(Conv2D)	(None, 28, 28, 512)	2359808
**block4_pool**	(MaxPooling2D)	(None, 14, 14, 512)	0
**block5_conv1**	(Conv2D)	(None, 14, 14, 512)	2359808
**block5_conv2**	(Conv2D)	(None, 14, 14, 512)	2359808
**block5_conv3**	(Conv2D)	(None, 14, 14, 512)	2359808
**block5_pool**	(MaxPooling2D)	(None, 7, 7, 512)	0

Appended Layers	(type)	Output Shape	Param #
**model_1**	(Model)	(None, 7, 7, 512)	14714688
**global_average_pooling2d_1**	(Global Average Pooling)	(None, 512)	0
**dense_1**	(Dense)	(None, 502)	257024
**batch_normalization_1**	(Batch	(None, 502)	2008
**activation_1**	(Activation)	(None, 502)	0
**dense_2**	(Dense)	(None, 667)	334834
**batch_normalization_2**	(Batch	(None, 667)	2668
**activation_2**	(Activation)	(None, 667)	0
**dense_3**	(Dense)	(None, 1)	668
