# Supplementary material for: Deep learning-based selection of human sperm with high DNA integrity
Source: Commun Biol. 2019 Jul 3;2:250. doi: 10.1038/s42003-019-0491-6 (PMC6610103; doi:10.1038/s42003-019-0491-6)
Supplement: Supplementary file 2 — Reporting Summary [file 42003_2019_491_MOESM2_ESM.pdf]

natureresearch

Corresponding author(s): David Sinton

Last updated by author(s): 05/14/2019

Reporting Summary

Nature Research wishes to improve the reproducibility of the work that we publish. This form provides structure for consistency and transparency in reporting. For further information on Nature Research policies, see Authors & Referees and the Editorial Policy Checklist.

Statistics

For all statistical analyses, confirm that the following items are present in the figure legend, table legend, main text, or Methods section.

n/a	Confirmed	
☐	☒	The exact sample size (*n*) for each experimental group/condition, given as a discrete number and unit of measurement
☐	☒	A statement on whether measurements were taken from distinct samples or whether the same sample was measured repeatedly
☐	☒	The statistical test(s) used AND whether they are one- or two-sided<br>*Only common tests should be described solely by name; describe more complex techniques in the Methods section.*
☐	☒	A description of all covariates tested
☐	☒	A description of any assumptions or corrections, such as tests of normality and adjustment for multiple comparisons
☐	☒	A full description of the statistical parameters including central tendency (e.g. means) or other basic estimates (e.g. regression coefficient) AND variation (e.g. standard deviation) or associated estimates of uncertainty (e.g. confidence intervals)
☐	☒	For null hypothesis testing, the test statistic (e.g. *F*, *t*, *r*) with confidence intervals, effect sizes, degrees of freedom and *P* value noted<br>*Give P values as exact values whenever suitable.*
☒	☐	For Bayesian analysis, information on the choice of priors and Markov chain Monte Carlo settings
☒	☐	For hierarchical and complex designs, identification of the appropriate level for tests and full reporting of outcomes
☐	☒	Estimates of effect sizes (e.g. Cohen's *d*, Pearson's *r*), indicating how they were calculated

Our web collection on statistics for biologists contains articles on many of the points above.

Software and code

Policy information about availability of computer code

Data collection	Image data were collected via confocal microscopy to produce large tiff images. These images were then cropped and rotated to select individual cells using the opencv-python package.
Data analysis	The convolutional network framework was written in Python v3.6 using Keras v2.1.5 over Tensorflow v1.8.0. We utilize the VGG16 convolutional network framework developed for Keras. Bayesian optimization using gaussian processes was performed via gp_minimize function of scikit-optimize v0.4 (Python v3.6). All supplementary data analysis was performed in Python v3.6 with matplotlib, numpy, pandas, scipy, and common packages.

For manuscripts utilizing custom algorithms or software that are central to the research but not yet described in published literature, software must be made available to editors/reviewers. We strongly encourage code deposition in a community repository (e.g. GitHub). See the Nature Research guidelines for submitting code & software for further information.

Data

Policy information about availability of data

All manuscripts must include a data availability statement. This statement should provide the following information, where applicable:

- Accession codes, unique identifiers, or web links for publicly available datasets
- A list of figures that have associated raw data
- A description of any restrictions on data availability

The datasets generated during and/or analyzed during the current study are available in the figshare repository, https://figshare.com/articles/Deep_learning-based_selection_of_human_sperm_with_high_DNA_integrity/8124932

Field-specific reporting

Please select the one below that is the best fit for your research. If you are not sure, read the appropriate sections before making your selection.

☒ Life sciences ☐ Behavioural & social sciences ☐ Ecological, evolutionary & environmental sciences

For a reference copy of the document with all sections, see nature.com/documents/nr-reporting-summary-flat.pdf

Life sciences study design

All studies must disclose on these points even when the disclosure is negative.

Sample size	We utilize 6 donor sperm samples with 150, 111, 89, 73, 134, and 507 individual sperm cells, respectively. These sample sizes are limited by the confocal microscopy technique used to obtain high magnification since we bound the sperm cells to a 2D planar surface and optimized for coverage density to limit cell overlap.
Data exclusions	No data were excluded
Replication	The model performance remained consistent and reproducible when re-trained from scratch, with similar correlation and percentiles obtained for individual donors, as indirectly observed in the learning curve analysis. Such consistency is expected when training on the same images with the same model architecture. Given new image training data and different model architecture, the model weights and predictions may vary, but the performance outlined in this manuscript is indicative of general performance.
Randomization	Cells were chosen as the final test set via randomized stratification to ensure the highest randomization. When training the neural network, input images were chosen at random and augmented (as described in Methods) to further randomize the input images.
Blinding	Blinding was not relevant to our study

Reporting for specific materials, systems and methods

We require information from authors about some types of materials, experimental systems and methods used in many studies. Here, indicate whether each material, system or method listed is relevant to your study. If you are not sure if a list item applies to your research, read the appropriate section before selecting a response.

Materials & experimental systems

n/a	Involved in the study	
☒	☐	Antibodies
☒	☐	Eukaryotic cell lines
☒	☐	Palaeontology
☒	☐	Animals and other organisms
☒	☐	Human research participants
☒	☐	Clinical data

Methods

n/a	Involved in the study	
☒	☐	ChIP-seq
☒	☐	Flow cytometry
☒	☐	MRI-based neuroimaging
